# HIV viral load monitoring among key populations in low‐ and middle‐income countries: challenges and opportunities

**DOI:** 10.1002/jia2.25003

**Published:** 2017-11-24

**Authors:** Sheree R Schwartz, Matthew M Kavanagh, Jeremy Sugarman, Sunil S Solomon, Illiassou M Njindam, Kevin Rebe, Thomas C Quinn, Coumba Toure‐Kane, Chris Beyrer, Stefan Baral

**Affiliations:** ^1^ Department of Epidemiology Center for Public Health and Human Rights Johns Hopkins School of Public Health Baltimore MD USA; ^2^ Center for Public Health Initiatives University of Pennsylvania Philadelphia PA USA; ^3^ O'Neill Institute for National and Global Health Law Georgetown University Washington DC USA; ^4^ Berman Institute of Bioethics Johns Hopkins University Baltimore MD USA; ^5^ Department of Medicine Johns Hopkins School of Medicine Baltimore MD USA; ^6^ Anova Health Institute Cape Town South Africa; ^7^ Division of Intramural Research National Institutes of Health National Institute of Allergy and Infectious Diseases Bethesda MD USA; ^8^ Department of Bacteriology and Virology CHU Le Dantec Dakar Senegal

**Keywords:** HIV, viral load, key populations, sub‐Saharan Africa, Asia, implementation, epidemiology, surveillance

## Abstract

**Introduction:**

Key populations bear a disproportionate HIV burden and have substantial unmet treatment needs. Routine viral load monitoring represents the gold standard for assessing treatment response at the individual and programme levels; at the population‐level, community viral load is a metric of HIV programme effectiveness and can identify “hotspots” of HIV transmission. Nevertheless, there are specific implementation and ethical challenges to effectively operationalize and meaningfully interpret viral load data at the community level among these often marginalized populations.

**Discussion:**

Viral load monitoring enhances HIV treatment, and programme evaluation, and offers a better understanding of HIV surveillance and epidemic trends. Programmatically, viral load monitoring can provide data related to HIV service delivery coverage and quality, as well as inequities in treatment access and uptake. From a population perspective, community viral load data provides information on HIV transmission risk. Furthermore, viral load data can be used as an advocacy tool to demonstrate differences in service delivery and to promote allocation of resources to disproportionately affected key populations and communities with suboptimal health outcomes. However, in order to perform viral load monitoring for individual and programme benefit, health surveillance and advocacy purposes, careful consideration must be given to how such key population programmes are designed and implemented. For example, HIV risk factors, such as particular sex practices, sex work and drug use, are stigmatized or even criminalized in many contexts. Consequently, efforts must be taken so that routine viral load monitoring among marginalized populations does not cause inadvertent harm. Furthermore, given the challenges of reaching representative samples of key populations, significant attention to meaningful recruitment, decentralization of care and interpretation of results is needed. Finally, improving the interoperability of health systems through judicious use of biometrics or identifiers when confidentiality can be maintained is important to generate more valuable data to inform monitoring programmes.

**Conclusions:**

Opportunities for expanded viral load monitoring could and should benefit all those affected by HIV, including key populations. The promise of the increasing routinization of viral load monitoring as a tool to advance HIV treatment equity is great and should be prioritized and appropriately implemented within key population programmatic and research agendas.

## Introduction

Despite a global plateauing in HIV disease burden, the decline in HIV incidence and expanded coverage of antiretroviral therapy (ART), HIV is not equally distributed across populations 1. Given the biology of HIV and individual, network‐level and structural risk determinants for infection, key populations (KP) are disproportionately at risk for HIV infection 2, 3, 4, 5. KP include gay men and other men who have sex with men (MSM), transgender women, sex workers, people who inject drugs (PWID) and incarcerated populations 6. Furthermore, KP remain generally underrepresented in HIV treatment programmes 7. There has been an increased focus on the content and implementation of HIV prevention strategies to serve KP, with the goal of achieving individual‐ and population‐level benefits 6. Condom provision, tailored HIV testing and counselling, and needle and syringe exchange programmes are examples of programmes that have been implemented for KP 8. While such interventions are critical, it is increasingly clear that greater attention to the HIV treatment needs of KP living with HIV is necessary to promote equitable access to healthcare and to change the course of the epidemic 9.

Access to viral load monitoring represents one strategy that can inform the implementation of programmes to address the specific treatment needs of KP. Specifically, routine viral load monitoring can serve as a metric of optimal adherence to ART and an indicator of potential ART resistance, thereby serving as a means of gauging treatment response. From an individual perspective, knowledge of viral load is a powerful tool for focusing on personal health since attaining and maintaining an undetectable viral load can motivate ART adherence by indicating to individuals that their regimen is working. As such, viral load monitoring is among the most powerful HIV prevention tools available 10.

Apart from individual‐level benefits, viral load monitoring can also be a population‐level HIV surveillance tool and a measure of programmatic success 11. These opportunities warrant further exploration as they can facilitate the evaluation of the global response to HIV.

Despite these potential benefits, the path to increased viral load monitoring among KP faces challenges, including the usual barriers of cost and limited laboratory capacity in low‐resource settings, as well as logistical and ethical hurdles unique to KP which are explored below 12. It is important to consider operational challenges and potential solutions, so that the benefits of this individual and public health intervention might be realized by those most affected by HIV.

In this paper, we explore opportunities along with associated cautions related to data interpretation and ethical implementation of viral load monitoring among marginalized KP in low‐ and middle‐income settings. We consider the potential benefits and challenges from programmatic, population and public health advocacy perspectives. Case studies selected from our collective work in West Africa, Southern Africa and South Asia provide illustrative examples (Table 1).

**Table 1:** Case studies of key populations viral load monitoring opportunities and challenges

Population	Country	Methods & illustration of viral load utility	Advancements	Challenges	Source
PWID, MSM	India	Serial cross‐sectional respondent driven sampling Population‐level surveillance	Prevalence of viraemia is the closest correlate of HIV incidence in the community Community viral load as a marker of epidemic trends	Viral suppression data is from research studies, which suggest that treatment and viral load monitoring must be provided alongside other CBO services due to gaps in linkages to referral centres	Solomon *et al*. 23. Mehta *et al*. 14. McFall *et al*. 53.
FSW	South Africa	Programmatic data and cross‐sectional respondent driven sampling Demonstration of programme effectiveness Indication of selection biases from use of programme data	Programmatic data from the urban centre of Hillbrow demonstrate higher rates of viral suppression among FSW participating in the programme than corresponding clinic data from the broader population	Programme data may mask population‐level disparities in treatment initiation and viral suppression among FSW not engaged in care Data collected through respondent driven sampling in Johannesburg suggested much lower viral suppression; 81% of FSW were not on ART and uncontrolled viral load would thus be even higher	Program data Wits Reproductive Health Institute *(Direct Correspondence, F. Venter)* University of California in San Francisco, Anova Health Institute, and WRHI 54
MSM, FSW	Cameroon	Implementation science and programmatic data Community‐based specimen collection	Viral load monitoring can be performed through integrated, community‐based programmes which collect specimens within the community	Lack of point‐of‐care diagnostics are a challenge; blood work for viral load monitoring is sent to reference laboratory, where results often take 45–60 days to be returned Results are directly communicated with patients for confidentiality purposes; however, this makes tailored counselling by case managers and peer educators difficult as they do not receive the results directly	CHAMP program data *(Direct correspondence I. Mfochive Njindam)*
Transgender FSW	South Africa	Programmatic data Impact of community‐based programmes on viral load	Clinician has taken HIV treatment and viral load monitoring services to a local NGO space in Cape Town Laboratory results are provided individually before support meetings No patients were previously linked to HIV treatment, many now virally suppressed	Absence of point‐of‐care diagnostics due to small scale of services Data sent to reference laboratory which requires patient names and sex which may not match patients’ identity	Anova Health Institute's Health4Men program data *(Direct correspondence K. Rebe)*
MSM	Nigeria	Cohort study Advocacy	Viral load can be an objective marker of the impact of stigma and discriminatory policies	Loss to follow‐up higher among men not engaged in care, potentially leading to overestimation of viral suppression	Schwartz *et al*. 52. Chauraut *et al*. 33.

PWID, people who inject drugs; MSM, men who have sex with men; CBO, community‐based organisation; FSW, female sex worker; ART, antiretroviral therapy; LTFU, loss to follow‐up. Case studies present work from authors or collaborators.

## Discussion

### Viral load monitoring as a health surveillance tool for KP

At the programmatic level, viral load monitoring can provide data regarding HIV treatment access, uptake and effectiveness. Low rates of viral suppression within geographic areas, hotspots, specific age or other subgroups may indicate a need for enhanced programming efforts or structural interventions 13. For example, HIV cascade analyses can highlight where individuals fall out of the HIV treatment cascade 14, 15, 16. Furthermore, identifying subgroups of individuals who are at increased risk of not achieving viral suppression and offering more intensive services or differentiated care to them may help to improve health outcomes, thereby targeting resources at those who most need them.

Given the significant risks of HIV transmission among members of KP with unsuppressed viral replication, programmatic efforts should be directed at implementing services tailored to the specific needs of diverse populations of KP who generally share structural determinants of risk including marginalization, and at times, mobility 17, 18. Culturally and clinically competent services, which foster improved treatment coverage and sustained adherence are necessary to improve health outcomes for KP 19, 20. Failure to do so can result in individuals at high risk of onward HIV transmission not accessing healthcare due to perceived or experienced stigma from non‐sensitized health providers 7, 21. To this point, however, most programmes have measured success based on services delivered to KP rather than on the impact or effectiveness of those services. As demonstrated through South African data, viral load offers a metric for programme effectiveness (Table 1).

From a population perspective, viral load data collected through integrated HIV biobehavioral surveys can help describe local epidemics and trends, as well as the population attributable fraction of HIV that could potentially be addressed through more effective KP programming 11, 22. Data from MSM and PWID in India suggest that the prevalence of detectable viraemia is a strong surrogate of HIV incidence, and thus may be an important programme evaluation as well as HIV surveillance tool (Table 1) 23. Similarly, assessment of community viral load may provide insights of epidemic trends in the population 23, 24, 25.

Caution must be taken, however, when using viral load data for HIV surveillance. Unsuppressed viral load can be used as a reason to blame KP for the epidemic 26. That is, there is the risk for unsuppressed viral loads to exacerbate stigma already associated with particular sexual and/or drug use practices if KP are seen as “bridge” populations potentiating transmission to the “general population”. Notably, these same HIV‐related determinants are generally not only stigmatized but often criminalized, both of which undermines treatment retention and adherence and thus reinforce poor outcomes 27, 28, 29, 30.

Additionally, selection biases, who is overrepresented and underrepresented in viral load data and how this relates to exposure to treatment, may affect understanding of epidemic trends within KP. There are multiple opportunities to reach KP with viral load monitoring, however, each approach has its own advantages and disadvantages (Table 2). For example, programmatic data tend to oversample people engaged in the programme, which may in turn overestimate overall engagement in care and treatment compared to sampling methodologies that achieve greater sampling depth and breadth across networks 31, 32. Thus, as noted in the South African case study (Table 1), high rates of viral suppression from non‐representative programme data may mask inequities amongst KP by overlooking those who are unengaged in care and treatment.

**Table 2:** Methods for sampling key populations (KP) for viral load monitoring

Sampling methods	Advantages	Disadvantages
Key population programme service delivery	KP‐identifiable and viral loads returnable using programmatic resources Blood draws can be collected in the community where KP are more easily reached	Sample includes those engaged in services and underrepresents those not engaged in care Data may include duplicates if biometrics are not utilized
Clinic data and national registries	Data longitudinal, assuming individuals are retained in care	Difficult and often impossible to identify KP through clinic data or national registries Sample includes those engaged in services and under‐represents those not engaged in care
Social network‐based recruiting, such as respondent driven sampling or snowball sampling	Methods can reach those not engaged in care Results may be generalizable to underlying population of interest If serial cross‐sectional studies are conducted, can ascertain insight into changes over time in terms of KP viral suppression	Difficult to verify that individuals truly belong to KP Need to account for recruitment methods, which may not be possible for subanalyses such as viral suppression due to breaks in chains since not all individuals enrolled will be living with HIV
Venue based sampling	Efficient recruitment method Community‐based viral load monitoring may reach those not engaged in care	May be difficult at certain venues to verify that individuals truly belong to KP Individuals who do not attend venues are not represented and may be substantively different from those that do In the absence of point‐of‐care diagnostics, returning results to individuals may be challenging Individuals may be recruited at multiple sites, potentiating duplicate enrolments if biometrics are not utilized

Furthermore, longitudinal KP programme or surveillance data from low‐ and middle‐income countries remain scarce. As observed in the Nigerian example (Table 1), those retained in services are likely very different in terms of viral suppression outcomes to those who are lost to follow‐up. Although this is true in any programme, consistent marginalization and extensive mobility of KP may contribute to poorer retention in care among KP, thus resulting in greater biases in viral load suppression rates due to differential loss to follow‐up among KP 33. Attempts to ascertain clinical outcomes of individuals lost to follow‐up from national registries or laboratory data may be limited by the inability of health monitoring and information systems to link patient data across clinics or geographic areas, particularly if, for confidentiality purposes, those initially accessing care in KP‐specific programmes were tracked through unique identifiers, which are deliberately not linked to a government‐issued ID nor national health records. Serial cross‐sectional viral load monitoring through repeat network‐based sampling methods studies, such as respondent driven sampling, or time‐space sampling may be used for population‐level HIV surveillance purposes and can circumvent some of the aforementioned problems 34.

### Considerations around implementation models for KP viral load monitoring in low‐resource settings

Despite the potential promise of viral load monitoring among KP in resource‐limited settings, implementation involves critical logistical and ethical concerns. These include where and how to reach KP in order to obtain specimens, how to return laboratory results to individuals, how to ensure treatment support, whether the frequency of viral load monitoring for populations at high risk of onward HIV transmission should follow or exceed national guidelines, how to protect anonymity and confidentiality of those engaged in activities prohibited by law, and how to safely integrate laboratory and clinical data between service delivery programmes and national health registries. Implementation experience of viral load monitoring for KP living with HIV in Cameroon highlights some of these challenges (Table 1).

KP may be easiest to reach in community settings; however, viral loads are typically done within clinical facilities. Furthermore, KP programmes typically refer those living with HIV to standard ART clinics that may not be sensitized to providing KP‐competent services, thereby resulting in substantial drops in linkage to care following HIV diagnosis 35, 36, 37, 38. Decentralized models which offer HIV testing, ART provision and management including viral load monitoring, STI screening and treatment, and TB treatment in a stigma‐free venue would likely have better HIV service outcomes for KP 8. These decentralized models providing care at community‐based organisations (CBOs) or mobile clinics can harness the potential of point‐of‐care viral load diagnostics as they become available or employ dried blood spots sent to reference laboratories 39.

Increasing focus on differentiated service delivery models which adapt care to patient needs and preferences present particular opportunities for KP. For example, adherence clubs, CBO‐based HIV services and mobile services could all become venues for viral load monitoring 40. Moreover, the nature of KP‐dedicated programmes could support increased focus on quality, potentially resulting in greater clinical utilization of viral load results in patient management. For instance, KP who use drugs or alcohol often experience suboptimal adherence and consequently higher treatment failure, prompting the need for more frequent viral load monitoring among these groups in order to identify treatment failure early, further preventing the development and onward transmission of resistant strains 21. In cases where KP‐specific training and services exist, the quality is often high and the potential for these programmes to effectively provide ART care is great 41.

### Biometrics to support individual viral load monitoring among KP

Viral load monitoring implementation challenges further include those related to data sharing and individual follow‐up. Given the mobile nature of KP, as individuals transition between research, KP‐specific service delivery and national treatment programmes, there is a need to consider how to streamline viral load monitoring rather than reinforcing parallel, duplicate systems. In order to protect the rights of participants engaging in activities that may be illegal and for whom the use of names or national IDs may increase risks, KP programmes often use unique identifiers, such as aliases or identification numbers. These unique identifiers may be generated by the client and provider based on a set of questions only the client can answer. Furthermore, in transgender populations, sex and legal names on official identification documents may not match the patient's gender or used name (Table 1). However, unique identifiers can complicate patient‐level tracing both within programmes and between different parts of the health system.

Biometric identifiers hold the potential to overcome some of these challenges by providing a technological solution that is easy to implement and able to be secured and encrypted. However, a biometric identifier is typically indelible, which can present significant human rights concerns for KP in some contexts 42. Ethical and human rights considerations suggest a need for close attention to security and identifying settings where biometrics may pose more risk than reward. Somewhat different considerations apply depending upon context.

First, in the healthcare delivery setting, medical records that track viral load can help ensure patients are retained in care and that both clinicians and individuals can act on viral load information – results that do not reach the patient or are not used to optimize treatment decisions provide no benefit. A unique identity signifies a unique person who warrants care and protection 43. Here, development of a biometric identified health record used by KP and non‐KP alike in the health system, integrated with KP‐service delivery, provides clear benefit to individuals. However, identifying specific individuals as engaged in criminalized activities creates data that can be exploited by governments and ill‐intentioned individuals to threaten the wellbeing of KP. Examples include police raids on a LGBT‐friendly clinic in Uganda and the recent government mandated closing of community‐based HIV organisations serving MSM in Tanzania, data breaches of electronic KP patient records, and evidence that some health workers share confidential information 44, 45, 46, 47. To enhance privacy, the data linked to biometrics should be limited to the minimal extent necessary for the intended purposes. In such records, there is rarely a need to identify individuals as KP. In addition, measures such as identifying clinics by numbers rather than names may help to improve confidentiality and privacy. Measures to add encryption, record biometric data like fingerprints as codes rather than as images, and boost data security are also essential 48. Guidelines on data access should be developed that make unauthorized use legally punishable. Taking these steps and consulting with KP communities before introduction of technology is critical.

Second, research and surveillance on viral suppression among KP ideally includes measures to avoid duplicate participants, for which some have suggested biometric identifiers or KP‐identifiable medical records. Sound data about a population subgroup is a first step in identifying a group‐level need for care 43. HIV surveillance alone, however, does not provide individual‐level benefit and potentially exposing individuals to harm in the process is ethically unjustifiable. Constructing databases of identifiable KP presents significant human rights concerns, heightened further by the insecurity of many databases and the potential for rights violators to use them to identify members of stigmatized KP. In addition, the very act of collecting biometrics, particularly fingerprints, can instil fear in criminalized groups, which is likely to bias participation. Therefore, while in less hostile settings biometrics may be appropriate, in environments where there is significant criminalization and stigma related to particular sexual and drug use behaviours, it is advisable to continue the use of anonymizing unique identifiers for research and surveillance. This may require duplicate sample collection for personal health records that are not KP identified, but the benefits are worth the added costs.

### Viral load monitoring as an advocacy tool

Viral load data can play a pivotal role in advocacy for human rights 49. The burden of unsuppressed viral load, coupled with the population attributable fraction estimates, can be used to demonstrate the need for services, support equitable allocation of resources, and evaluate progress toward realization of the right to health for KP 50. Comparing viral suppression between KP and the broader population in different countries should help to identify inequities and identify focus areas for programming. This has been done on a broader scale to advocate for specific geographic areas or age groups and can similarly be applied among KP 51.

Furthermore, viral load monitoring data can be used to demonstrate the impact of policies or to advocate for an intervention. For example, in Nigeria, viral load data served as an objective metric to demonstrate the negative impact of healthcare‐related stigma on HIV treatment outcomes 52. These data were also used as an indirect measure of the effect of a discriminatory legal policy on health outcomes and thus served as an important public health advocacy tool 52.

## Conclusions

As routine viral load monitoring becomes the standard of care for patients living with HIV, it offers important benefits for individuals, programmes and communities. There are many opportunities for expanded use, and new technologies have the potential to be “leapfrog” advances addressing operational, structural and programmatic challenges in HIV service provision. It is essential that KP are not forgotten in innovative methods for scale‐up of ART programmes nor the rollout of routine viral load monitoring. These advances must result in benefit for KP individuals and the communities most affected by HIV. An AIDS‐free generation is simply impossible without the achievement of equity for KP and the communities in which they live and work. The promise of viral load monitoring as a tool to advance HIV treatment equity is great, and should be realized with all due urgency and careful implementation.

## Competing interests

The authors have no competing interests to declare.

## Authors’ contributions

All of the authors (SRS, MMK, JS, SSS, IMN, KR, TCQ, CTK, CB and SDB) contributed to the conceptualization and writing of the manuscript.
